# The Proposed Royal Commission

**Published:** 1913-08-02

**Authors:** 


### THE PROPOSED ROYAL COMMISSION.

Last week we drew attention, incidentally, to the very weighty and powerful appeal issued to the medical profession in the *Lancet* by Sir Malcolm Morris, in which was urged the necessity for a Royal Commission upon Venereal Diseases. Since our article was penned, a public pronouncement in favour of Sir Malcolm's suggestion has been signed by forty of the most eminent members of the medical profession, headed by Sir Thomas Barlow. We feel it a public duty to speak out in favour of the appointment of such a Commission, and to express the hope that the Prime Minister will favourably consider the matter. Last Tuesday he was questioned in the House of Commons about it, and confined himelf to a non-committal answer. In the circumstances this was as much as could be expected; Mr. Asquith has had little time of late to make himself familiar with the subject and the altered conditions brought about by recent research, but with the Parliamentary recess coming on he may perhaps have more leisure to study the brief which it must be the business of the medical profession to present to him.

It cannot be too clearly emphasised that the proposals of Sir Malcolm Morris, to which the Presidents of both Royal Colleges have lent their support, are purely and simply for investigation of the extent of the evil and for recommendations as to how it may be combated. Many years ago one particular legislative attempt to mitigate the Secret Scourge was made, and then abandoned. Feeling ran high between two small bodies, those who were in favour of that particular legislation and those who were against it; but the enormous majority of the population knew and cared nothing about the matter at all. Now matters are different in many ways. Several of our ablest contemporary novelists and dramatists have depicted the awful horrors which ensue, not now and then, but every day and everywhere, from venereal diseases, horrors in which the innocent suffer with the guilty, and infants in arms are spared not. Then, again, the education of the public in matters of hygiene is proceeding apace. Tuberculosis is being attacked under State auspices; the infectious fevers are made a matter for the local authorities to deal with. So gradually the apathy of the public in regard to this other pestilence is wearing thin; and the time has come to drop the conspiracy of silence which has too long hindered the forces of health and hygiene. It is therefore greatly to be hoped that a really strong Royal Commission will be appointed, and that pending its investigations there shall be nothing done or said by hot-heads on either side of the ancient controversy to embitter their opponents or to bemuse the public. Quite probably the discoveries of the last five years may have altered the strategy of the campaign against this widespread evil so fundamentally that the parties to the old disputes may find themselves left with nothing to quarrel about. But at least we do need authoritative guidance as to what the future plan of campaign is to be. Finally, we would express our admiration of the courage which the *Morning Post* has displayed in publishing the letter of the forty doctors; other organs of the press have had the pluck to follow this lead, and we congratulate them too.

